# Cardiac sympathetic overdrive, M2 macrophage activation and fibroblast heterogeneity are associated with cardiac remodeling in a chronic pressure overload rat model of HFpEF

**DOI:** 10.3389/fphar.2024.1364758

**Published:** 2024-05-27

**Authors:** Fengjiao Sun, Ling Yuan, Zi Wang, Xiaoxue Cui, Nan Lv, Ting Zhang, Yan Zhang, Jun Cai

**Affiliations:** ^1^ Cardiovascular and Cerebrovascular Drugs Research and Development Center, Tianjin Institute of Medical and Pharmaceutical Sciences, Tianjin, China; ^2^ Department of Pathology, Tianjin Institute of Medical and Pharmaceutical Sciences, Tianjin, China; ^3^ Pharmaceutical Analysis Laboratory, Tianjin Institute of Medical and Pharmaceutical Sciences, Tianjin, China; ^4^ Traditional Chinese Medicine Formulation Research Laboratory, Tianjin Institute of Medical and Pharmaceutical Sciences, Tianjin, China; ^5^ Department of Cancer Pharmacology, Tianjin Institute of Medical and Pharmaceutical Sciences, Tianjin, China

**Keywords:** sympathetic overactivation, M2 macrophage activation, pressure overload hypertrophy related fibroblasts, heart failure with preserved ejection fraction, fibrosis

## Abstract

Heart failure with preserved ejection fraction (HFpEF) is a multifaceted pathogenesis disease and the exact mechanisms driving HFpEF have not been completely elucidated. Pressure overload hypertrophy (POH) related fibroblasts and M2 macrophages in HFpEF myocardium have been recently identified and are now of great interest. Sympathetic overdrive has also been implicated in HFpEF. This study is designed to dynamically observe the potential roles of aforementioned mechanisms in pathological remodeling and cardiac dysfunction in chronic PO rats. Surgical constriction of the abdominal aorta was used for induction of HFpEF. Echocardiography, electrocardiogram, hemodynamic measurement, hematoxylin and eosin staining, Masson staining, immunohistochemistry and immunofluorescence were performed to assess the changes in heart dysfunction, cardiac remodeling and driving mechanisms at different time points (2, 18, 24 weeks). The PO induced HFpEF model was well established, which was confirmed by the persistent increase in carotid artery systolic and diastolic blood pressure, and left ventricle hypertrophy at the corresponding postoperative stage. Meanwhile, PO hypertrophy gradually developed into HFpEF, associated with QT and QTc intervals prolongation, normal systolic (EF was maintained at >50%) but impaired diastolic function (increasing LVEDP and LV -dP/dt_min_, abnormal E/A ratio), increased myocytes size, and observed relatively slight inflammatory infiltration but robust reactive fibrosis. IHC staining further confirmed that macrophages (CD68) but not neutrophils (MPO) or T cells (CD3) accounted for a predominant proportion of infiltrating cells. Mechanistically, we found that the infiltrating macrophages in the heart expressed high levels of CD206 which was simultaneously adjacent to POH fibroblasts appeared to overexpression of α-SMA in PO rats at late stages. Interestingly, we distinguished two different POHF sub-populations during PO induced HFpEF development, according to non overlapping signals of α-SMA and PDGFRα/β proteins. Additionally, PO led to a pronounced exaggeration in sympathetic fibers at all time points. These findings suggest that the establishing model here begins with cardiac sympathetic overdrive, subsequently along with immune cells especially M2 macrophage accumulation and fibroblast heterogeneity at later stages is associated with the development of cardiac maladaptive remodeling and diastolic dysfunction thus further progression to HFpEF.

## Introduction

Despite dramatic advances in heart failure (HF) research setting, HF with preserved ejection fraction (HFpEF), a subtype of HF, remains a major clinical problem and affects approximately 50% of all HF cases (DeBerge et al., 2019). For decades, the pathogenesis and pharmacological treatment for HF with reduced EF (HFrEF) are well established. HFpEF is understudied compared with HFrEF, and the pathophysiological mechanisms of HFpEF have yet to be clearly defined. Thus far, no known therapeutic agents that specifically improve HFpEF are available (DeBerge et al., 2019).

The HFpEF differs from the HFrEF, not only functionally or because the initial stimuli that drives HFpEF are in the absence of cardiac cell death or focal cardiac injury, but also because it is considered that the emergence of an activated fibroblast population triggering pathological matrix remodeling that contributes to the progression of HFrEF and HFpEF are largely distinct (DeBerge et al., 2019; Oatmen et al., 2020). Specifically, myofibroblasts, the classical reparative fibroblasts, are widely believed as the principal effector cells responsible for fibrosis and wound healing in HFrEF. By contrast, the activation and expansion of an abnormal fibroblast phenotype, referred to as the pressure overload hypertrophy (POH) fibroblast which is similar to apoptosis-resistant cancer associated fibroblasts (CAFs), is emerging as a key player in driving progression from POH to HFpEF by transforming the normal myocardial stroma to an abnormal fibrotic structure (Moore-Morris et al., 2015; Oatmen et al., 2020). Subsequently, the progressive and insidious myocardial fibrosis leads to stiffening of the myocardium, and eventually impairs diastolic performance, the hallmark of HFpEF.

In addition to POH fibroblasts represent attractive therapeutic targets, the profibrotic role of M2 macrophage has also shown great promise as novel effective cell for cardiac fibrosis in the setting of HFpEF (Westermann et al., 2011; Glezeva et al., 2015; Hulsmans et al., 2018; DeBerge et al., 2019). Cardiac macrophages expand in humans and animals with HFpEF, which has been reported overexpression of pro-fibrotic genes such as transforming growth factor (TGF)-β and interleukin (IL)-10 and further directing cardiac fibroblast activation and subsequently leading to excess collagen deposition (Westermann et al., 2011; Hulsmans et al., 2018). Beyond the heart, circulating increased classical and alternative monocytes in HFpEF patients are also responsible for left ventricle (LV) diastolic function. Intriguingly, serum from HFpEF individuals incubated healthy monocytes preferentially acquired M2 macrophage features (Glezeva et al., 2015). In this regard, recent work in an experimental model demonstrated that specific deletion of IL-10 in macrophages was expected to improve diastolic function (Hulsmans et al., 2018). These events suggest that the pro-fibrotic role of M2 like macrophages could be deleterious in the HFpEF setting. Together, both POH fibroblast phenotype and M2 like macrophage may be very strongly implicated in HFpEF pathogenesis. Moreover, sympathetic nerve activity has been shown to be exaggerated in HFpEF, which is known to accelerate maladaptive remodeling and cardiac dysfunction in HF (Badrov et al., 2021). Although above multiple mechanisms could be associated with the process of HFpEF development, better understanding the mechanism is often limited by the lack of appropriate animal models.

Hypertension has been identified as a major risk factor for HFpEF which frequently results from POH (Oatmen et al., 2020). Abdominal aorta constriction (AAC) is a well established animal model for inducing POH owing to hypertension and it has shown to mimic different types of HF (Cantor et al., 2005; Tan et al., 2021). The present study was undertaken to explore the time-course changes of hemodynamic, systolic and diastolic function, as well as cardiac pathology and fibrosis. Furthermore, the role of M2 macrophage infiltration, fibroblast activation and sympathetic nerve control were also dynamically investigated in the chronic PO induced HFpEF model.

## Materials and methods

### Establishment of the HFpEF model

All protocols for experiments were conducted in accordance with the Guide for the Care and Use of Laboratory Animals of the National Institutes of Health and approved by the animal ethics committee of Tianjin Institute of Medical and Pharmaceutical Sciences (no. IMPS-EAEP-Z-KJ20148-01). The objects were male Sprague Dawley rats (weighting 230–250 g) and HFpEF model was set up by abdominal aorta constriction as previously described (Sun et al., 2014; 2019). Briefly, the rats received general intraperitoneally anesthesia with sodium pentobarbital (50 mg/kg). After midline incision, the abdominal aorta between the superior mesenteric artery and right renal artery was exposed. Then a blunted 7-gauge needle was placed over the aorta and a 4–0 surgical suture was used to tie securely around the vessel and needle. The blunt needle was withdrawn from the ligature and the wound was closed. Sham-operated rats served as controls and underwent identical surgery except for the ligation of the abdominal aorta. Each rat received penicillin immediately post surgery. PO model animals and age-matched sham animals were used at 2-, 18- and 24- week time points after the procedure. Fifteen animals were included in each group for each time point. The survival rate was 100% in sham rats and the total mortality rate was 10% in PO-operated rats throughout the course of the experiment.

### Echocardiography

After rats were anaesthetized with isoflurane in pure oxygen, echocardiography was performed in the rats using a 21-MHz transducer attached to a Visualsonics Vevo 2100 system (Canada) as previously described (Sun et al., 2019). For systolic function, M-mode measurement was used to determine ejection fraction (EF), LV mass at parasternal long axis view. For diastolic function, pulsed wave Doppler mode was obtained to assessed early and atrial waves (E and A) and E/A ratio of the mitral valve velocity at the apical four-chamber view.

### Electrocardiographic and hemodynamic measurements

Electrocardiographic and hemodynamic evaluations were performed as previously described (Sun et al., 2018; Sun et al., 2019). In brief, the rats were anaesthetized with pentobarbital sodium (50 mg/kg, ip). Subsequently the lead II surface electrocardiogram (ECG) was acquired using the ML870 PowerLab System (AD Instruments, Australia). The consecutive ECG data were recorded for 10 min per animal. The following parameters including heart rate (HR), QT interval and rate-corrected QT (QTc) interval were analyzed. Then the polyethylene catheter (PE-50) was used to insert into the right common carotid artery to obtain the systolic, diastolic and mean blood pressures (SBP, DBP and MBP) variables. For LV hemodynamic, the polyethylene tubing was then advanced into the LV cavity, and the parameters, including LV systolic pressure (LVSP), LV end-diastolic pressure (LVEDP) and the maximum rates of LV pressure increase and decrease (LV + dP/dt_max_ and -dP/dt_min_, respectively) were automated recorded. All these data were analyzed using LabChart 7.3.7 software (AD Instruments, Australia).

### Histopathological examination

The hearts were excised and fixed with 10% formaldehyde, then embedded in paraffin wax and cut into 3–5 μm thick longitudinal sections. Histological changes and myocardial fibrosis were evaluated by hematoxylin-eosin (H&E) staining and Masson trichrome staining (BSBA-4079A, Beijing Zhongshan Golden Bridge Biotechnology Co., Ltd., Beijing, China) respectively as previously described (Sun et al., 2019). The collagen distribution appeared blue. Five micrographs were captured from each section at 200 × magnification and collagen volume fraction [CVF, CVF (%) = interstitial or perivascular collagen fiber area/total area of the image × 100%] was calculated to assess LV interstitial and perivascular fibrosis. To examine cell size, wheat germ agglutinin (WGA) staining was carried out. Slides were incubated with WGA-FITC labeled antibody (1:100, L4895, Sigma Aldrich) for 30 min at room temperature, and cells were counterstained with DAPI to visualize the nuclei in blue. Digital images were collected by optical microscope or fluorescence microscopy (Nikon Corporation, Tokyo, Japan), and analysis was performed using ImageJ 1.46r software.

### Immunohistochemistry and immunofluorescence

Immunolabeling was performed on deparaffinized sections to examine various immune cell types, cardiac fibroblasts and nerve fibers as the previous study did (Elia et al., 2021). To block endogenous peroxide activity, the sections were incubated in 3% H2O2 solution for 10 min and antigen retrieval was performed using a citrate-based antigen unmasking solution or Tris-EDTA buffer. The heart sections were then blocked with 5% bovine serum albumin for 20 min at room temperature and incubated with the following primary antibodies: polyclonal rabbit anti-CD3 (1:500, 17617-1-AP, Proteintech), polyclonal rabbit anti-myeloperoxidase (MPO, 1:500, 22225-1-AP, Proteintech), polyclonal rabbit anti-CD68 (CD68, 1:500, 28058-1-AP, Proteintech), polyclonal rabbit anti-mannose receptor antibody (CD206, 1:2500, ab64693, Abcam), monoclonal mouse anti-alpha smooth muscle actin (α-SMA, 1:1000, NBP2-33006, Novus), monoclonal rabbit anti-platelet derived growth factor receptor alpha + beta antibody (PDGFRα/β, 1:150, ab32570, Abcam), polyclonal rabbit anti-tyrosine hydroxylase (TH, 1:500, 25859-1-AP, Proteintech), overnight at 4°C. For immunofluorescence analysis, double staining of α-SMA and PDGFRα/β or CD206 in heart longitudinal sections was performed. The α-SMA (1:200 or 1:500, NBP2-33006, Novus) and PDGFRα/β (1:150, ab32570, Abcam) were applied for labeling POH fibroblast, whereas CD206 (1:1000, ab64693, Abcam) was used to identify M2 macrophage. Washed slides were incubated with the appropriate secondary antibodies separately, anti rabbit-HRP (1:500, abs20040, Absin), anti mouse-HRP (1:500, abs20001, Absin), anti rabbit-Alexa Fluor^®^488 (1:500, 4412S, CST), anti mouse-Alexa Fluor^®^594 (1:500, 8890S, CST), anti rabbit-Alexa Fluor^®^594 (1:500, 8889S, CST), anti mouse-Alexa Fluor^®^488 (1:500, 4408S, CST). And IHC staining was followed by using a DAB substrate kit for color development. To visualize nuclei, sections were staining with hematoxylin for IHC staining or DAPI for immunofluorescence respectively. All images were observed and captured using an optical microscope or Nikon Ni-U fluorescence microscope. The protein expression level in IHC staining was measured using ImageJ (version 1.46r). In each sample, five fields of view were selected in LV and the average value was determined for analysis. And the percentage of positive staining area was calculated by the equation below: total area of brown-yellow granules/total area of image × 100%.

### Statistical analysis

All the data were analyzed by SPSS 19.0 software (IBM Corporation, Armonk, NY, United States). The student’s unpaired *t*-test was used for comparisons between two groups. And multiple comparisons were examined using One-way analysis of variance, followed by Dunnett’s test. Differences with *p*-value <0.05 were considered as statistically significant.

## Results

### Echocardiographic observation

**FIGURE 1:**
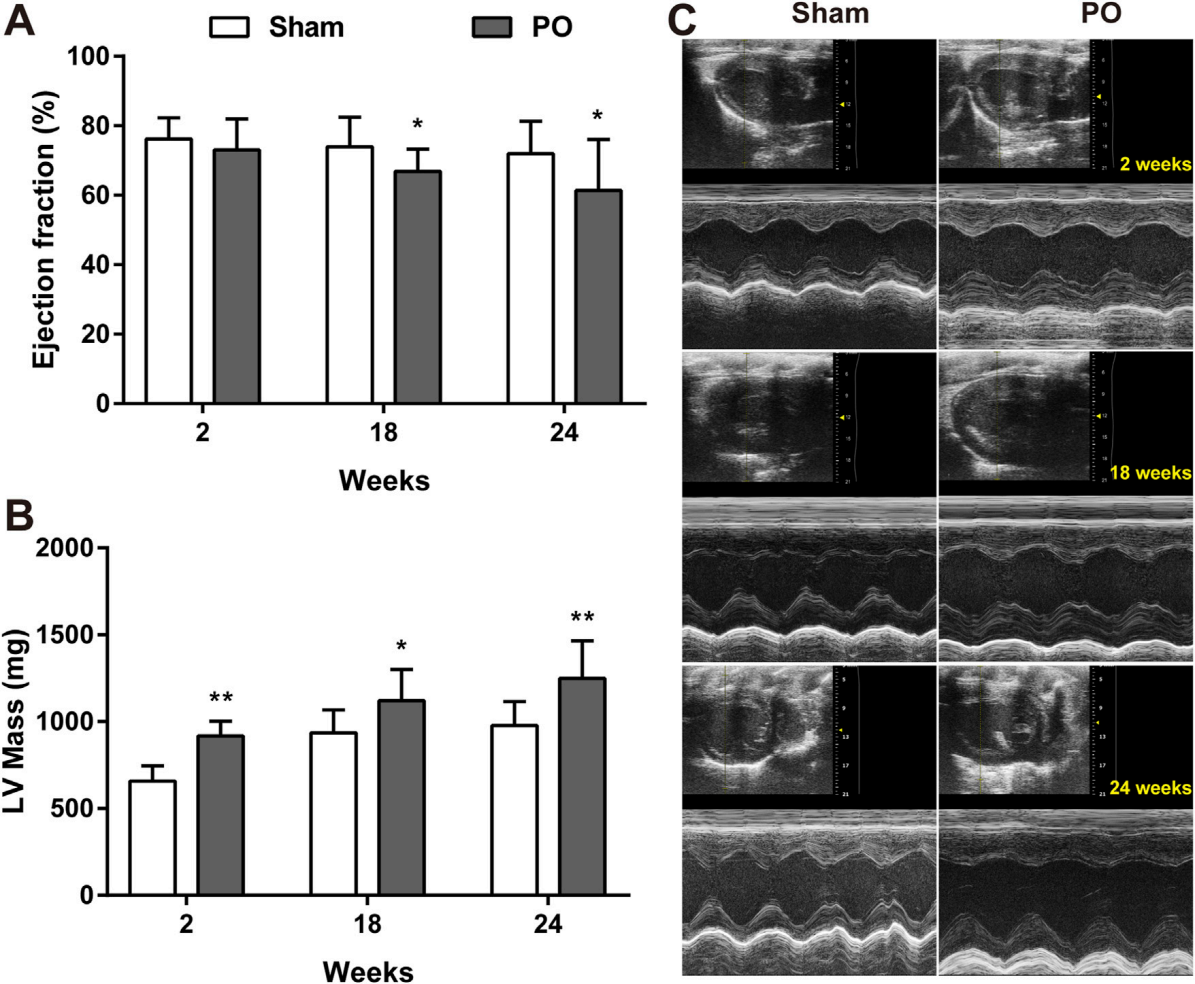
Serial echocardiography for LVEF and LV mass of each group. LV systolic function presented as LVEF **(A)**, LV mass **(B)** measured from the echocardiography and the representative serial M-mode echocardiographic tracings were exhibited at 2, 18, 24 weeks after operation **(C)**. Values were presented as mean ± SD, *n* = 15/group. * *p* < 0.05 and ** *p* < 0.01 vs. Sham group.

**FIGURE 2:**
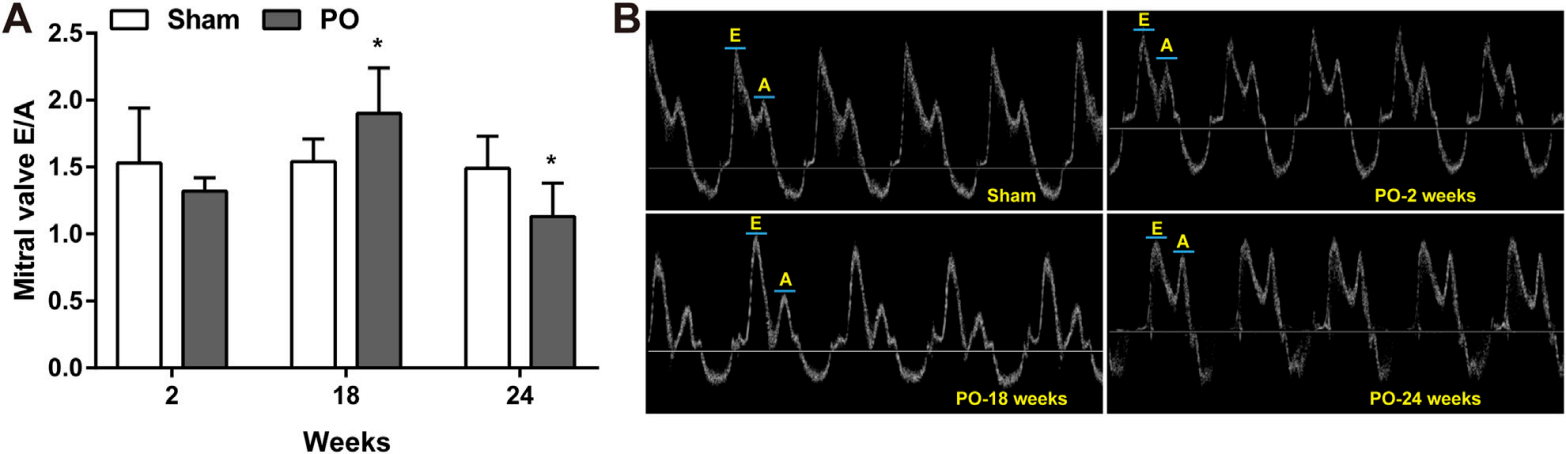
Serial echocardiography for E/A ratio at different time points. Cardiac diastolic function index E/A ratio expressed as the ratio of peak velocity of early to late filling of mitral inflow **(A)** and representative images of A and E wave **(B)** were shown. Values were presented as mean ± SD, *n* = 8–10/group. * *p* < 0.05 vs. Sham group.

The serial echocardiographic results were shown in Figure 1 and Figure 2. Although PO animals exhibited a slight but significantly decreased EF at 18 and 24 weeks, the LV systolic function was still preserved since the LVEF values above 60% at corresponding time points (Figure 1A). In PO rats, a noted increase in LV mass was initially found at 2 weeks, and this trend continued throughout the remainder of the time-course (Figure 1B). Furthermore, by contrast to Sham control, the E/A value in PO rats was not significantly altered at 2 weeks but was statistically significant higher at 18 weeks and lower at 24 weeks (Figure 2A), indicating LV diastolic dysfunction.

### Blood pressure and ECG parameters

**FIGURE 3:**
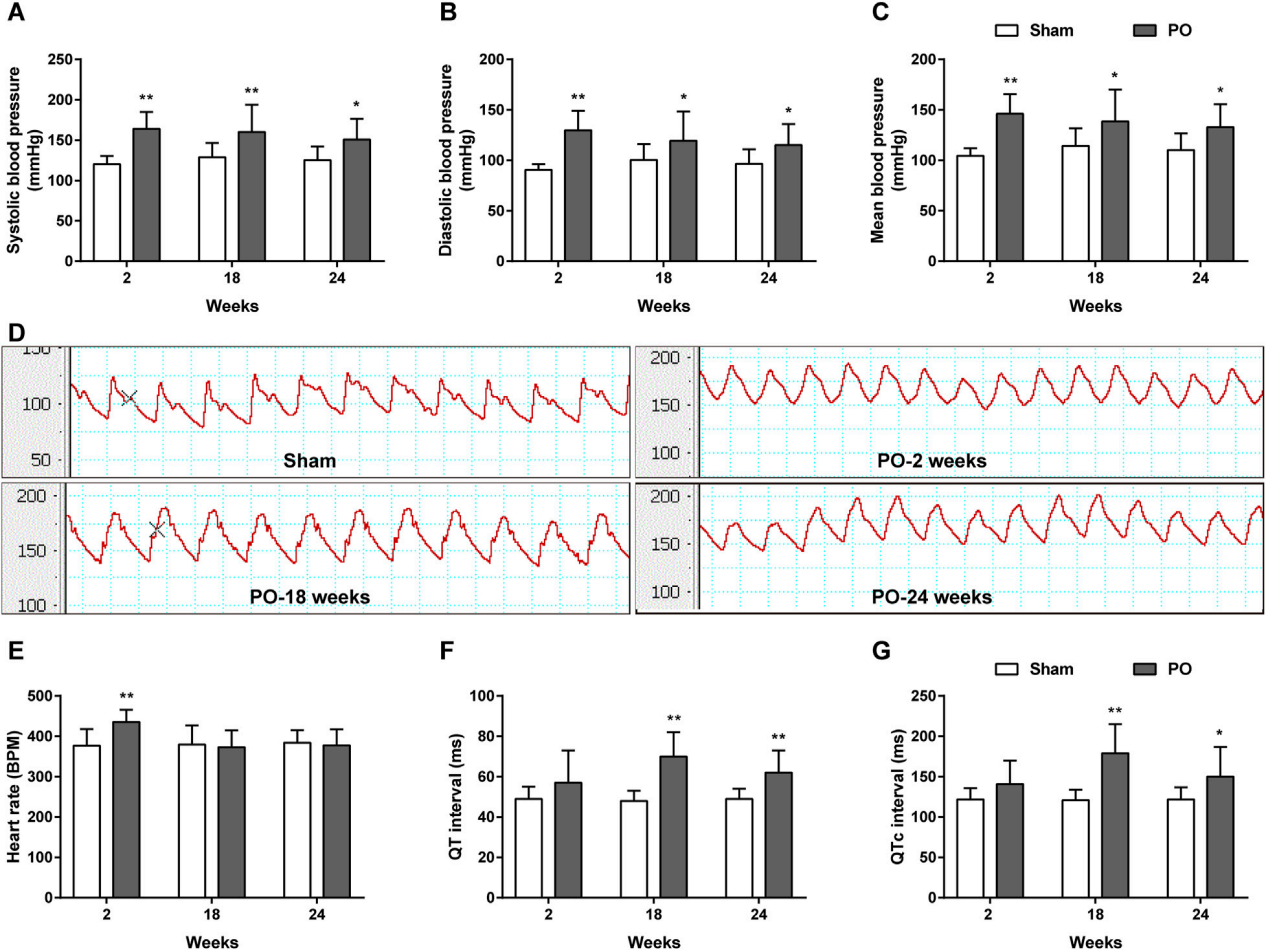
The dynamic patterns of carotid blood pressure and ECG parameters. Summary data for SBP **(A)**, DBP **(B)**, MBP **(C)**. **(D)** Representative BP traces in Sham and PO rats. Summary data for HR **(E)**, QT **(F)** and QTc interval **(G)**. Values were presented as mean ± SD, *n* = 10/group. * *p* < 0.05 and ** *p* < 0.01 vs. Sham group.

As shown in Figures 3A–D, the carotid artery pressure including SBP, DBP and MBP in PO models significantly increased as early as 2 weeks and continuously maintained the growth trend as compared to Sham group at following time points. Increases in these parameters represented chronic pressure overload in PO animals after AAC operation. Figures 3E–G demonstrated a higher heart rate but no changes in QT, QTc internals in PO animals at 2 weeks relative to Sham group. As the disease progressed, PO rats had a longer QT, QTc internals than Sham rats at 18 and 24 weeks, indicating of ECG disturbances.

### Hemodynamic changes

**FIGURE 4:**
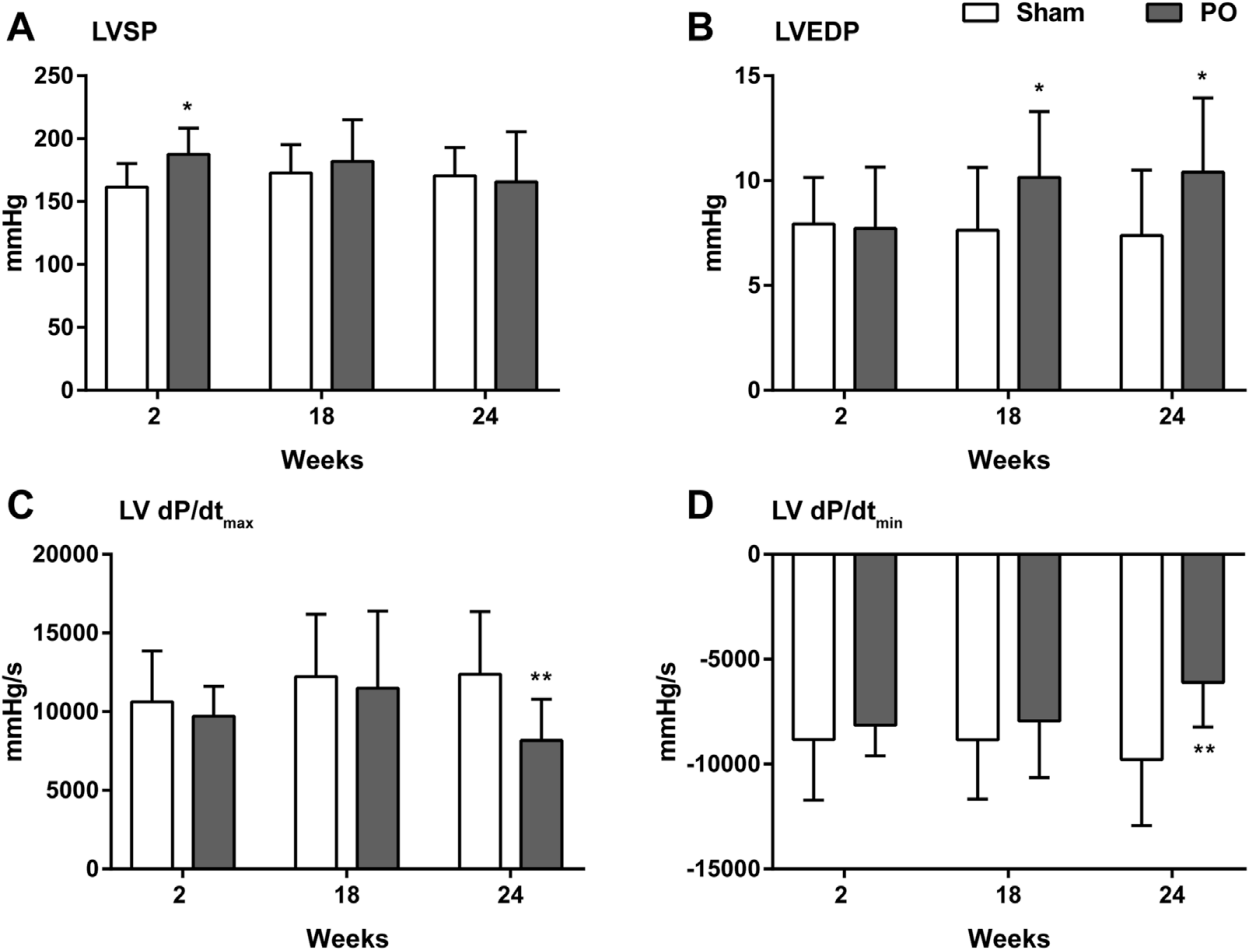
Changes in the hemodynamic parameters of each group. These data were shown for LV systolic pressure **(A)**, LV end-diastolic pressure **(B)**, the maximum increase in the LV pressure **(C)** and the maximum decrease in the LV pressure **(D)**. Values were presented as mean ± SD, *n* = 8–10/group. * *p* < 0.05 and ** *p* < 0.01 vs. Sham group.

PO rats were assessed hemodynamically, and the results were given in Figure 4. At 2 weeks of PO, LVSP was temporarily increasing (Figure 4A) whereas changes of LVEDP, LV + dP/dt_max_ and -dP/dt_min_ were not apparent. In agreement with previous echocardiography results, LV decreased contraction was confirmed by significantly declining of +dP/dt_max_ at 24 weeks (Figure 4C). Similarly, impaired LV relaxation was evidenced by a continuous elevation of LVEDP from week 18 onwards and increasing in -dP/dt_min_ at 24 weeks (Figures 4B, D).

### Morphological findings and fibrosis

**FIGURE 5:**
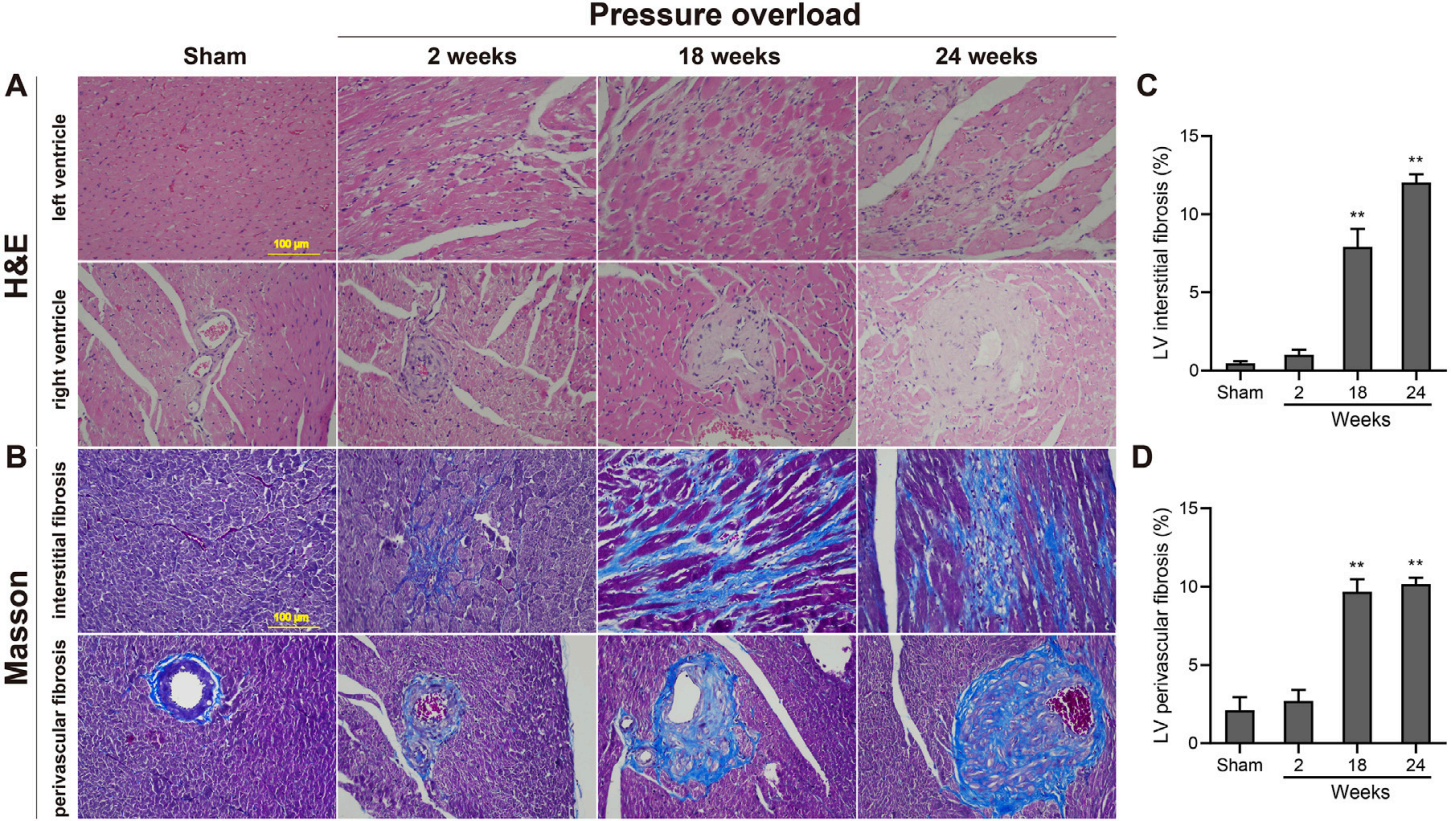
Morphology and fibrosis observations of each group following chronic pressure overload detected by hematoxylin-eosin and Masson staining. **(A)** Representative photomicrographs of slight inflammatory cell infiltration within the LV myocardium and severe artery structural changes within the RV area. **(B)** Representative Masson-stained heart sections from each group and quantification of the LV interstitial fibrosis **(C)** and perivascular fibrosis **(D)** in each group. Scale bar, 100 μm. Values were presented as mean ± SD, *n* = 5/group. ** *p* < 0.01 vs. Sham group.

**FIGURE 6:**
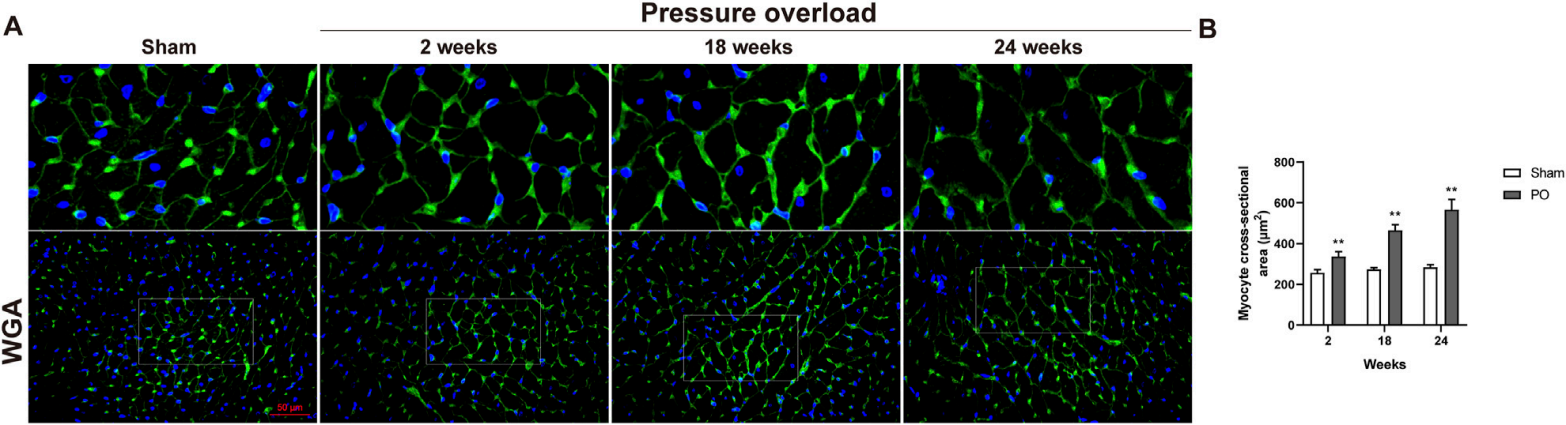
The effect of abdominal aorta constriction on cardiomyocyte hypertrophy. **(A)** Representative images of heart cross sections by WGA staining. **(B)** Quantitative data of myocyte cross sectional areas. Scale bar, 50 μm. Values were presented as mean ± SD, *n* = 5/group. ** *p* < 0.01 vs. Sham group.

As illustrated in Figure 5, histological analysis of H&E-stained heart sections revealed that there was mild increase in artery intima-media thickness within the right ventricle (RV) myocardium in PO rats at 2 weeks. As PO progressed, increase in artery wall thickness developed to moderate degree which was accompanied by narrowing of the vascular lumen diameter. By the end of week 24, the structural damage of arteries in RV area was aggravated to a severe extent (Figure 5A). Apart from these, inflammatory cell infiltration within the LV myocardium was sparse at 18 weeks and developed into a mild degree at 24 weeks respectively (Figure 5A). Masson trichrome staining revealed that interstitial and perivascular collagen deposition especially located in deep intramyocardium at the LV lateral wall shown no change at 2 weeks, a significant enlargement at 18 weeks and was more intensely at 24 weeks after AAC procedure (Figures 5B–D). As expected, the PO induced enlargement of cardiomyocytes confirmed by WGA staining was coincident with above-mentioned increase in LV mass at all time points observed (Figures 6A, B), suggesting an obvious myocyte hypertrophy in rats from the PO groups.

### Immune cell infiltration and M2 macrophage activation

**FIGURE 7:**
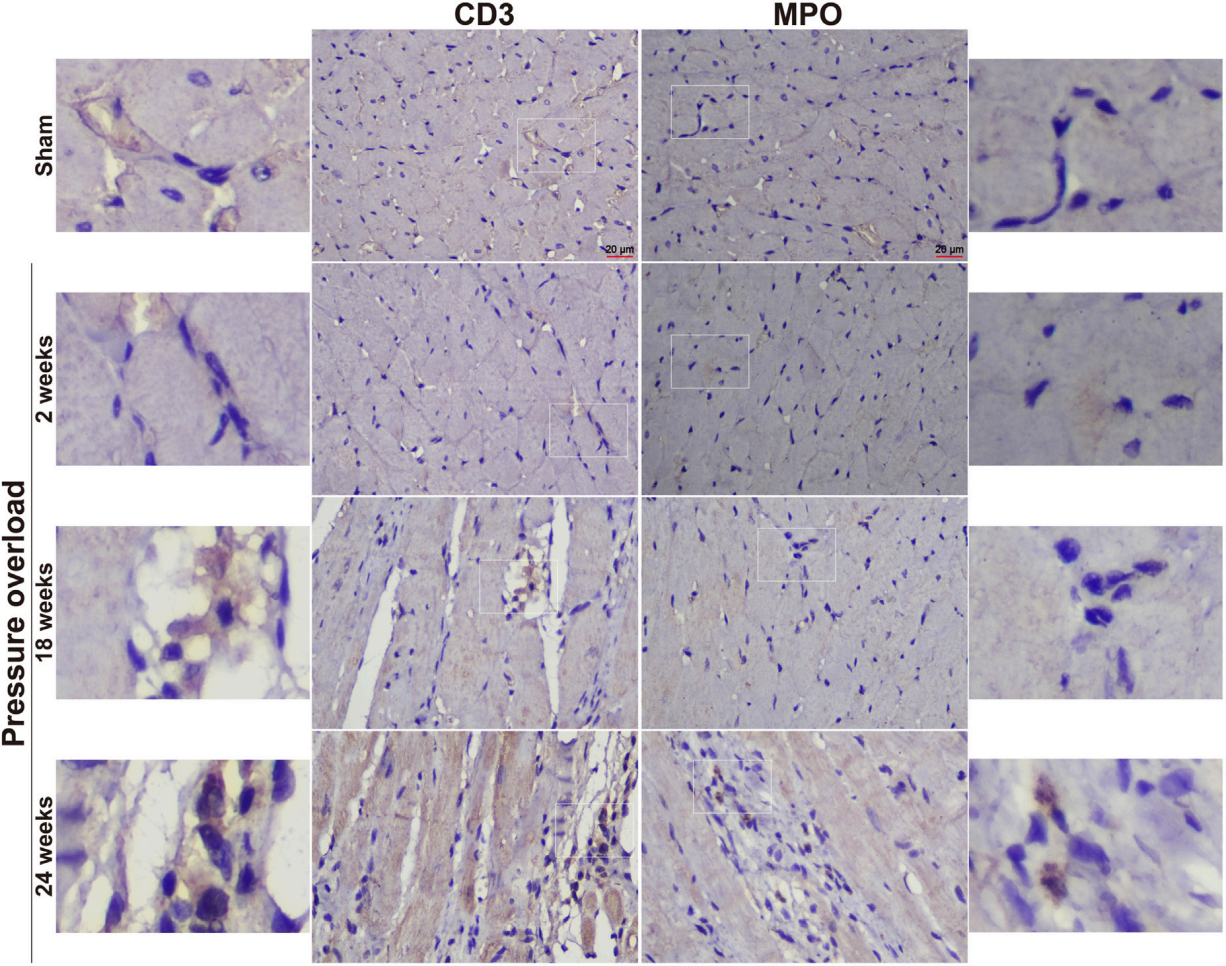
Representative staining of CD3 and MPO protein expression in the heart tissue. Scale bar, 20 μm.

**FIGURE 8:**
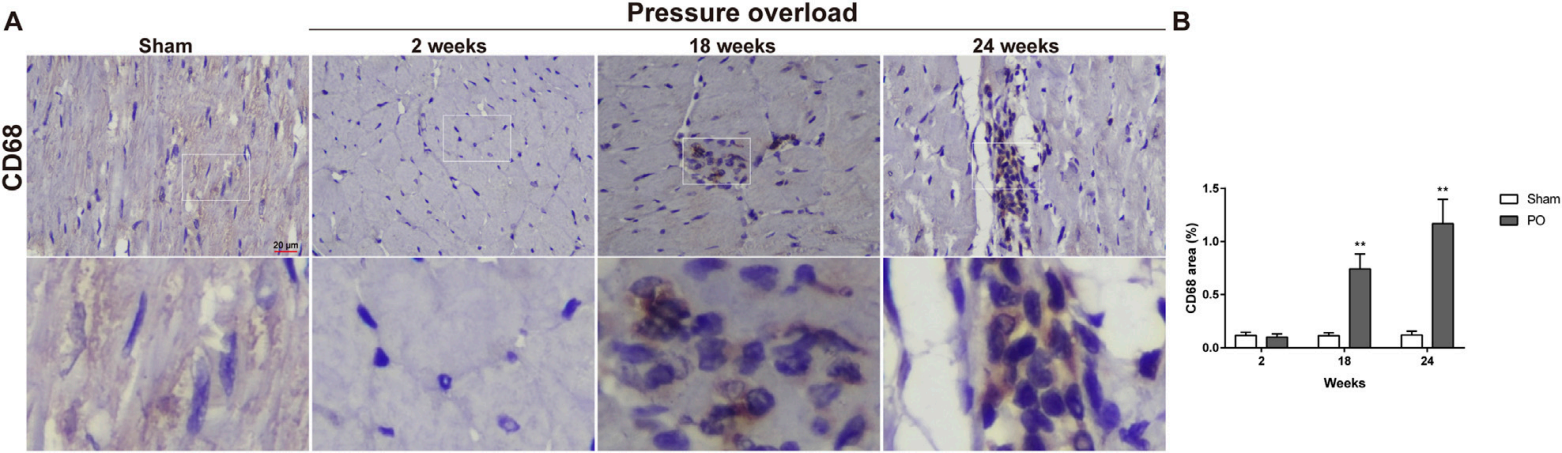
The number of heart interstitial macrophages was increased in PO rats. **(A)** Representative images of CD68 positive cells in LV at each group. **(B)** Quantification of CD68 expression in the heart. Scale bar, 20 μm. Values were presented as mean ± SD, *n* = 5/group. ** *p* < 0.01 vs. Sham group.

**FIGURE 9:**
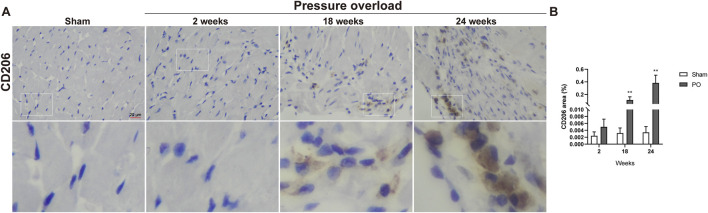
The number of M2 macrophages was elevated in PO hearts. **(A)** Representative photomicrographs of heart sections stained with CD206. **(B)** Bar graph resulted from quantitative analysis of CD206, an indicator marker of M2 macrophages. Scale bar, 20 μm. Values were presented as mean ± SD, *n* = 5/group. ** *p* < 0.01 vs. Sham group.

Immunostaining was performed to identify cells of T cells (CD3), neutrophils (MPO) and monocyte/macrophage lineage (CD68) in both Sham and PO rats as shown in Figure 7 and Figure 8. Immunohistochemistry of PO specimens exhibited that CD3 and MPO positive cells were scarce until the 24 weeks end point (Figure 7). Quantification of the staining demonstrated that macrophage infiltration in PO rats was significantly elevated compared with Sham controls at 18 and 24 weeks (Figures 8A, B). Furthermore, CD206, a marker for M2 macrophage phenotype, was subsequently verified to be consistently expanded at 18 and 24 weeks (Figures 9A, B). These results suggested M2 macrophage activation, an observation that has not been described in PO induced HFpEF animal models.

### Cardiac fibroblast activation

**FIGURE 10:**
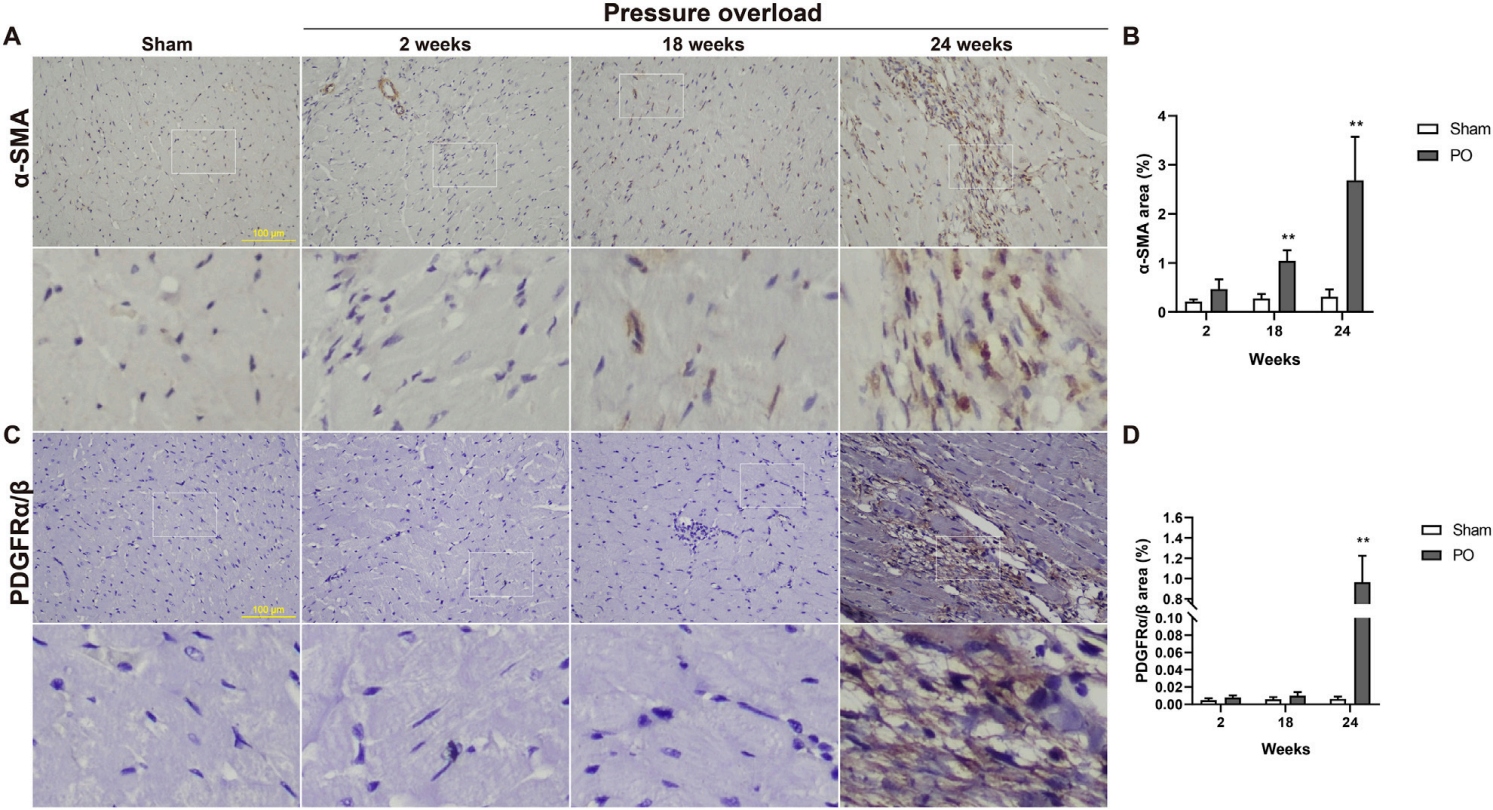
Immunohistochemical staining of activated fibroblasts within the myocardium of Sham or PO rats was exhibited. **(A,C)** Representative images of α-SMA and PDGFRα/β positive cells. **(B,D)** Quantitative analysis-derived histogram shown for α-SMA and PDGFRα/β, as markers for POH fibroblasts. Scale bar, 100 μm. Values were presented as mean ± SD, *n* = 5/group. ** *p* < 0.01 vs. Sham group.

**FIGURE 11:**
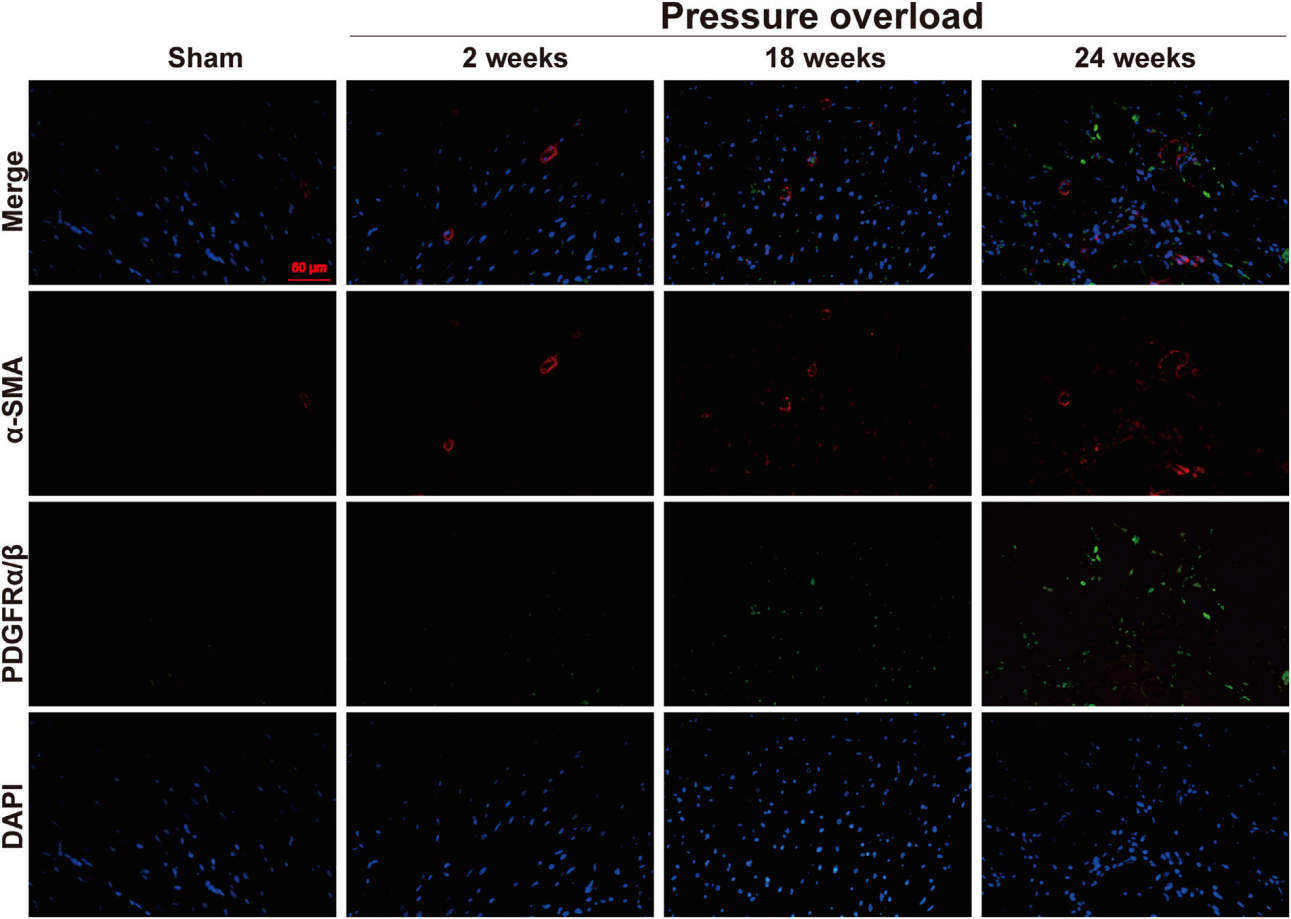
Representative views of α-SMA and PDGFRα/β *in situ* immunofluorescence of heart tissue. Blue: DAPI; green: PDGFRα/β; red: α-SMA; the green and red signals were not overlapped. This suggested two different subsets of POHFs. Scale bar, 50 μm.

Both α-SMA and PDGFR are vital markers for cardiac fibroblast activation. Here, expression of α-SMA in PO hearts was no significant changes firstly and then gradually increased during PO progress and finally reached a peak advanced to 24 weeks (Figures 10A, B). Similarly, PDGFRα/β expression did not appear to increase at 2 and 18 weeks; however, when PO developed to 24 weeks, PDGFRα/β positive cells extensively expanded in the interstitial space between cardiomyocytes (Figures 10C, D). In parallel, we further performed immunofluorescence detection and observed that α-SMA and PDGFRα/β positive cells actually represented two different subsets of fibroblasts (Figure 11). Thus, immunofluorescence data confirmed cardiac fibroblast activation and demonstrated for the first time the existence of two distinct POHF sub-populations in PO induced HFpEF.

### α-SMA and CD206 immunofluorescence

**FIGURE 12:**
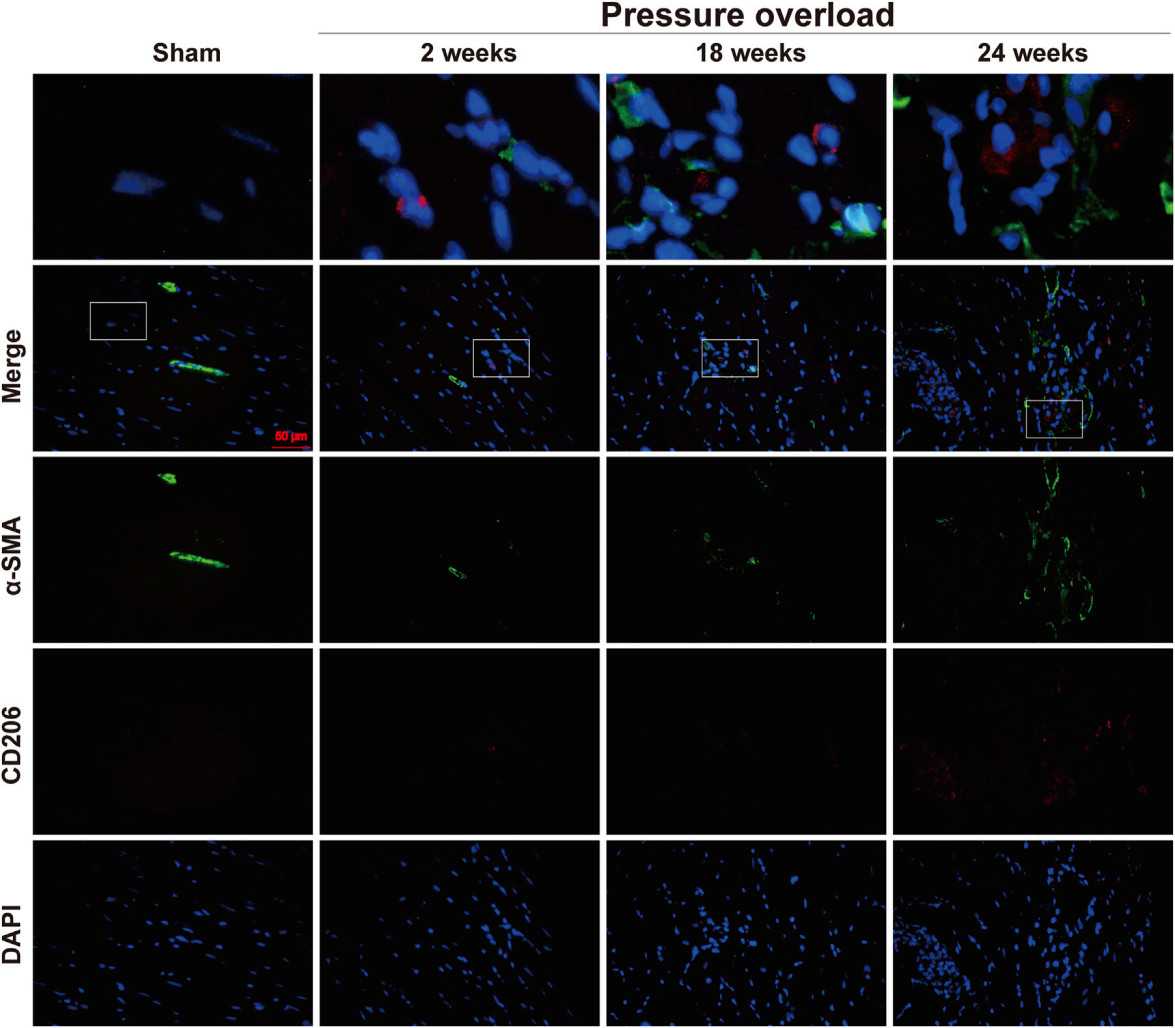
Representative immunofluorescence staining of close localization of α-SMA with CD206 in the myocardium of each group. Blue: DAPI; green: α-SMA; red: CD206; the merged images indicated a close association between M2 macrophages and neighboring activated fibroblasts. Scale bar, 50 μm.

Distribution of α-SMA and CD206 proteins was determined by co-immunofluorescence. As illustrated in Figure 12, positive α-SMA cells in Sham hearts were mainly presented in coronary vessels, whereas the appearance of α-SMA and CD206 in PO hearts was especially prominent in the myocardial interstitium at 18 and 24 weeks, confirming obvious activation and expansion of fibroblasts and M2 macrophages. The close localization of α-SMA and CD206 was further observed in interstitial areas, thus indicating that these changes in non-myocyte cells could be important in the progression to HFpEF.

### TH positive innervation

**FIGURE 13:**
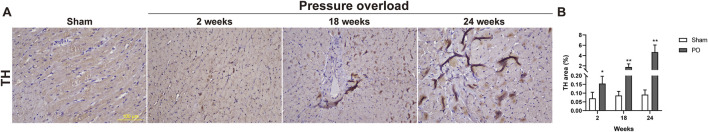
The density of sympathetic fibers was increased in myocardium of PO rats. **(A)** Representative images for TH positive expression. **(B)** Bar graphs derived from quantification TH + area. Scale bar, 100 μm. Values were presented as mean ± SD, *n* = 5/group. ** *p* < 0.01 vs. Sham group.

The TH fibers densities plotted as a regulating function of sympathetic nerve were measured and displayed in Figure 13. The LV in response to chronic pressure overload caused a significant elevation of TH fibers starting from as early as 2 weeks, and the amplitude for TH immunopositive fibers was sharply increased at late stages (Figure 13B). This result suggested an increase in cardiac sympathetic tone, the typical characteristic of HFpEF.

## Discussion

The risk factors for HFpEF are divergent, and hypertension is one of the most common chronic diseases which contribute to HFpEF. AAC is a clinically relevant animal model that mimics the pathophysiological changes in human hypertension since it can induce the progression from compensated hypertrophy to HF (Cantor et al., 2005; Tan et al., 2021). Here, SBP, DBP, MAP in PO rats increased at 2 weeks and sustained this trend over the 24 weeks period, suggesting that AAC resulted in a rapid hypertensive response as previously reported (Kuwahara et al., 2002; Kuwahara et al., 2004). Moreover, early and progressive increase in LV mass along with enlargement of myocyte size in our study indicated the early initiation and continuous development of pressure overload hypertrophy.

One of the most characteristic of sympathetic overactivation in either hypertrophic or failing heart is elevating HR (Voora and Hinderliter, 2018). Actually, in PO rats, HR was noted increased at 2 weeks indicating sympathetic activation at the beginning. However, as the disease progressed, HR had no change as compared to Sham control but ECG disturbances were further evidenced by the prolonged QT and QTc intervals duration at 18 and 24 weeks. Meanwhile, it has been widely accepted that the prolongations of QT and QTc internals play an important role in aggravating ventricle repolarization delay (Ramezani-Aliakbari et al., 2019), which could partially counteract the sympathomimetic effect on the heart and get involved in the unaltered HR at late stages as shown in our PO model.

Both invasive hemodynamic and noninvasive echocardiography have been employed most frequently for LV function evaluation (Wichi et al., 2007). Here, these two different techniques were jointly performed in our experiment to try to get a more convincing conclusion. In the hemodynamic perspective, LV function in our study appeared to be initially compensated at 2 weeks, as indicated by the increasing LVSP. This is in contrast to data at late disease stages, where LV function evidenced by attenuated + dP/dt_max_, confirming a systolic dysfunction; as well as increased LVEDP and elevated -dP/dt_min_, showing diastolic impairment. With regard to echocardiographic parameters, although systolic function was preserved in PO rats until the end of the experiment, there was a mild decline in LV contraction due to obviously decreased EF value. As expected, diastolic dysfunction was also represented by altered E/A ratios in PO model at 18 and 24 weeks. The decrease in the ratio E/A at 24 weeks could be interpreted as pseudo-normalization of diastolic function (Schnelle et al., 2018). Taken together, these observations were consistent with the vital criteria in establishing a diagnosis of HFpEF which exhibits LV diastolic dysfunction with a preserved LVEF.

A variety of surgical or pharmacological interventions have been used to establish HFpEF animal models. Unlike models (Kuwahara et al., 2002; Kuwahara et al., 2004; Tan et al., 2021) that stimulate an acute inflammatory response to promote LV functional impairment, our results were consistent with clinically relevant HFpEF model, with later onset and more slighter cardiac inflammation, slow development and prolonged course. Most strikingly, in this report, we identified heart-infiltrating macrophages were the main inflammatory cells and only scarce infiltrated T lymphocytes and neutrophils were found as late as 24 weeks after surgery. This was paralleled with predominantly of macrophages but few neutrophils infiltration in one recently published SAUNA-induced HFpEF mice model (Zhang et al., 2022). One hallmark of HFpEF is sustained systemic low-grade inflammation, which is usually resulted from the extra-cardiac comorbidities. It can also act as a drivers and eventually insult the cardiac tissue (Simmonds et al., 2020; Mesquita et al., 2021). Thus, in addition to inflammation in the heart, circulating inflammatory parameters is also recommended to assess the inflammatory state. More recently, some of the most compelling data indicated unchanged inflammatory cells infiltration in myocardium whereas the circulating inflammatory biomarker sST2 was largely increased in a metabolic syndrome associated HFpEF rat model. Therefore, it cannot be excluded that other circulating inflammatory markers, which were not tested here, could be strongly activated (Hubesch et al., 2022).

Macrophages, a type of innate immune cells, have high heterogeneity and multiple functions in polarizing into both pro-inflammatory M1 phenotype and anti-inflammatory M2 phenotype. These phenotypes play distinct and even opposite roles, and their uncontrolled activation promote heart injury and fibrosis in response to different environmental setting (DeBerge et al., 2019). In HFpEF patients, M2 like subsets have been shown to exert strong protumoral activities and contribute to the pathogenesis of heart fibrosis although M2 macrophages can promote cardiac repair in the context of myocardial infarction (MI) related HFrEF (Westermann et al., 2011; Glezeva et al., 2015; Hulsmans et al., 2018; DeBerge et al., 2019). However, the role of M2 phenotype in HFpEF animal models remains controversial, as one recent study identified increased M2 macrophage population in the 3 Hit mouse (Li et al., 2023) whereas other group have conversely reported their decrease in HFpEF hearts induced by uninephrectomy surgery and d-aldosterone infusion (Zhang et al., 2021). In this study, markedly increased numbers of M2 macrophages in PO induced HFpEF model were verified, and could be served as a new positive result in consistent with clinical patients with HFpEF.

An interesting finding in our PO induced HFpEF phenotype is that the presence of relative mild inflammatory infiltration is coincided with strongly elevated myocardial fibrosis (both interstitial and perivascular). Obviously, the increased collagen deposition in myocardium here cannot be explained completely by the activation of inflammatory process. In terms of non-myocyte cell types, activated fibroblasts expansion could also contribute to HFpEF development. In POH related HFpEF, fibroblasts can adopt an “active” state known as acquired apoptosis-resistant phenotype. This activation and proliferation process is associated with expression of a number of markers (Moore-Morris et al., 2015; Oatmen et al., 2020). Specifically, both α-SMA overexpression and expression of a constitutively activated PDGFRα are implicated in POH fibroblast proliferation and fibrosis (Moore-Morris et al., 2015; Ivey et al., 2019; Oatmen et al., 2020). Here, we highlight stromal heterogeneity during the progression of HFpEF by identifying two subsets of POHFs (α-SMA + or PDGFRα/β+). This result corresponded to diffuse LV fibrosis in HFpEF along with notable accumulation of CD206+ M2 macrophages. These findings were further strengthened by an additional dual IF staining, in which a close localization of α-SMA + fibroblast and CD206+ macrophage in heart tissues was observed. Given HFpEF as interstitial cancer has been proposed coupled with the known interactions between CAFs and tumor-associated macrophages (TAMs, a M2 phenotype) that are associated with stromal heterogeneity and immunosuppressive environment during tumor progression (Tromp et al., 2017; Oatmen et al., 2020; Yang et al., 2020), future work will be interesting to investigate the roles of the novel paradigm of cell to cell interactions in myocardial microenvironment remodeling and HFpEF development.

If the inflammatory reaction was now not pronounced in the preceding stage of HFpEF, then what was responsible for the LV remodeling? It is noteworthy that the long term profile of sympathetic nerve activation is also profoundly characterized. Some studies suggest that persistent β adrenergic activation as hallmark for sympathetic overactivation can mediate continuous increase in cAMP and further activating PKA to produce a cardiotoxic effect and our prior work has already published overstimulation of the PKA at 18 weeks in PO rats (Sun et al., 2019). Herein, our results shown that sympathetic hyperactivity had an early appearance (increased HR) at 2 weeks and became more pronounced confirmed by TH positive protein elevation with the long-lasting progression of hypertensive state. A large body of work has convincingly demonstrated both cardiac fibroblasts and virtually all immune cell-types express β adrenergic receptor (βAR), and these cells are involved in the pathological process of HF due to chronic sympatho-adrenergic overstimulation (Abboud et al., 2012; Fujiu and Nagai, 2013; Grisanti et al., 2016). Further clinical support evidence has shown that early hypertensive phases and established hypertensive states are characterized by increased sympathetic activity and sympathetic cardiovascular influences (Mancia and Grassi, 2014). Considering these results, it is likely that cardiac sympathetic nerve excess activation accompanying early hypertension has not only a initiating but also a sustaining component that may promote the recruitment of innate immune cells especially M2 macrophages and expansion of two different POHF sub-populations, which can further amplify the malignant remodeling of the heart.

In summary, we developed a long term PO induced model of HFpEF in rats characterized by diastolic abnormalities but preserved EF and LV remodeling including QT and QTc intervals prolongation, cardiomyocytes hypertrophy, fibrosis, inflammatory cell infiltration, and that is resemble to clinically observed changes in HFpEF patients. Based on temporal changes, it could be hypothesized that the presentation of M2 macrophages and two distinct POHF subsets, as well as the sympathetic fibers overexpression, all together strongly indicate that sympathetic overdrive initially at 2 weeks along with subsequently non-myocytes expansion at later stages constructs a microenvironment that promotes the pathophysiological cardiac remodeling during progression from hypertension stage to further HFpEF. This model could be useful for preclinical drug testing and basic research aimed at elucidating the mechanisms of preceding and established HFpEF.

## Data Availability

The raw data supporting the conclusion of this article will be made available by the authors, without undue reservation.
